# Effect of digital messaging on blood pressure control in general practice: observations from the BP@Home programme in wirral area

**DOI:** 10.1038/s41371-025-01072-y

**Published:** 2025-09-25

**Authors:** Antonios A. Argyris, David Baker, Paul Charnley, Liam Howe, Steven H. M. Lam, Gregory Y. H. Lip, Eduard Shantsila, Alena Shantsila

**Affiliations:** 1 Liverpool Centre for Cardiovascular Science at University of Liverpool, Liverpool John Moores University and Liverpool Heart and Chest Hospital, Liverpool, UK; 2 Heatherlands Medical Centre, Woodchurch, Cheshire, UK; 3 Healthy Wirral Partners, Wirral, UK; 4 Victoria Central Health Centre, Wallasey, UK; 5 Danish Center for Health Services Research, Department of Clinical Medicine, Aalborg University, Aalborg, Denmark

**Keywords:** Diseases, Hypertension

## Abstract

Home blood pressure (BP) monitoring (HBPM) is an established method for improved diagnosis and control of high BP. The BP@Home programme was launched in 2021 to support the use of HBPM. BP tele-messaging systems allow the digital transmission of HBPM readings back to healthcare providers, offering the potential to improve hypertension management. Aim of our study was to examine the enabling effect that implementing a new BP tele-messaging service had on hypertension metrics of an urban area. Through a new integrated BP text messaging system within GP platforms, practices from Wirral region, UK were able for the first time to contact their patients digitally to transmit their HBPM readings back to the practice. These readings were reviewed by the practice clinicians and patients received appropriate therapeutic recommendations. A total number of 10,010 patients were included in a 20-month period. The rate of HBPM per 1000 hypertensives ranged from 14.4–26.8 between different Primary Care Networks. BP control had been achieved in 64.3%. In linear regression analysis, use of HBPM was significantly associated with higher rates of new hypertension diagnosis [Beta coefficient (95% Confidence Interval (CI)): 0.11 (0.02–0.20), p = 0.021] and BP control [Beta coefficient (95% CI): 0.48 (0.40–0.56), p < 0.001]. In this community study using a BP tele-messaging implemented approach, system wide BP messaging was associated with an increase in new hypertension diagnoses and better control of hypertension. Future studies should focus on the role of digital BP messaging in reducing cardiovascular disease and improving clinical outcomes.

## Introduction

Cardiovascular diseases (CVD) are the leading cause of global mortality, leading to 17.9 million deaths in 2019 according to reports from World Health Organization [1, 2]. Arterial hypertension is the most important major, modifiable CVD risk factor [3, 4], accountable for over 10.8 million deaths annually worldwide [1, 4]. The prevalence of hypertension is rising, owing to lifestyle changes, the ageing of the population, as well as the increase of the obesity pandemic [5–7]. Results from a pooled analysis including 19.1 million adults indicated an increase in the number of patients with high blood pressure (BP), from 594 million in 1975–1.13 billion in 2015 and a global prevalence of 24.1% in men and 20.1% in women [6]. National studies in high-income countries revealed a much higher prevalence of hypertension, reaching 40% in men and 36% in women in the United Kingdom (UK) [8].

Awareness and treatment rates of hypertension have substantially increased in the last decades, much of the improvement taking place before the mid-2000s, and afterwards reaching a plateau between 40 and 80% [8]. In the UK, these numbers ranged from 67–70% for awareness and only 55–59% in treatment rates overall, with much lower rates in the younger age groups [8, 9]. Despite the availability of effective BP-lowering drugs and numerous randomized clinical trials demonstrating their ability to prevent CVD and lower mortality, the actual control rate of hypertension remains concerningly low[9–11]. Studies show that the implementation of effective hypertension management programs using currently available antihypertensive medications and effective patient monitoring can lead to very high rates of BP control [12]. In the UK, control rates were reported at 37% overall, with much lower control rates in younger patients [8, 9].

Current guidelines recommend the use of out-of-office BP measurements for diagnosis and management of hypertension [13, 14]. Home blood pressure monitoring (HBPM) is especially encouraged in terms of confirming diagnosis, identifying specific hypertension phenotypes and monitoring treatment response and refining risk stratification [15–17]. Studies have shown that increased utilization of HBPM can lead to higher rates of awareness and control of hypertension [18–20]. Recently, evolution of mobile technology, along with the COVID-19 pandemic, accelerated the adoption of remote BP monitoring strategies. Not only in the UK, but also globally, mobile health applications, including the BP@Home programme have been implemented to maintain hypertension control during health service disruptions (https://www.england.nhs.uk/ourwork/clinical-policy/cvd/home-blood-pressure-monitoring/) [21, 22]. BP telemonitoring is not regularly implemented in daily clinical practice in most countries, but digital health systems seem to offer a promising approach and the potential to improve hypertension management [23]. These systems offer remote monitoring and transmission of BP measurements to a predefined healthcare facility, leading to improved quality of the collected information and enabling the automated or manual feedback from a multidisciplinary clinical team [23].

However, there is a significant gap in knowledge regarding the feasibility, efficacy and long-term results of these strategies in different populations. The aim of our study was to assess the enabling effect that a new BP tele-messaging system had on the utilization of HBPM and the diagnosis and control of hypertension in an urban area of UK. This effect could be higher BP diagnosis and better control using these systems.

## Subjects and methods

The present study was an audit of routinely collected, fully anonymized data summaries.

### Population, study design and BP telemonitoring system

GP practices throughout the Wirral area, a mixed urban/suburban area in North West England, with high mobile phone and internet access, received funded licences to utilise the BP messaging aspect of an integrated Primary Care tele-messaging system already in use. The BP@Home messaging functionality is part of a broader telehealth platform commissioned by NHS, integrated into GP electronic systems and is available to participating practices across the UK. Patients used their own validated upper-arm oscillometric BP monitors at home. They were instructed by their GP practices to measure BP twice daily (morning and evening) for 4–7 consecutive days, following standardized protocols. Patients were then routinely contacted through mobile phone messages to submit their BP readings remotely. After completion of HBPM by the patients, their BP readings were transmitted to the corresponding physician through a designated platform. The platform used is a fully integrated system within GP practices with the capability to connect patients with their respective primary care facility, in terms of patient management, providing remote health care and incorporating data security measures. Through this platform, BP measurements performed at home from each patient were periodically transmitted via internet or mobile phone services, were reviewed by the physician and finally resulted in specific recommendations, in terms of lifestyle changes or adjustment of antihypertensive medication. Available data were extracted for the whole population and were categorized by primary care network (PCN). Two of the GP practices merged during the study period, so all data derived from these two practices were excluded from the analysis. Outcome measures were new hypertension diagnosis and BP control on the basis of SBP/DBP levels <140/90 mm Hg for patients <80 years or <150/90 mm Hg for patients ≥80 years old [24]. Although BP readings were obtained at home, we used office-based BP thresholds for determining control, in line with NICE NG136. This approach aligns with practice-level quality indicators and allows consistency across practices.

### Ethics approval and consent to participate

This is a service evaluation as part of the network hypertension pathway that was reviewed and approved via internal governance routes, specifically through Wirral Digital Primary Care Forum and Cheshire and Merseyside CVD Prevention Board. All methods were performed in accordance with the relevant guidelines and regulations. All participants provided informed consent to participate in this study.

### Statistics

Database management was performed using MS Excel and statistical analysis with SPSS version 28.0 (IBM, Chicago, Illinois, USA). A two-sided p < 0.05 was considered statistically significant. Continuous variables were expressed as mean ± standard deviation (SD) for normal data or median and interquartile range (IQR) for non-normal data; categorical variables were expressed as absolute values and percentage. Data were available for the total population and for different PCN. Data were plotted for the visualization of the monthly distribution of different outcome measures between different PCN. Poisson distribution analysis was employed for comparison of outcomes between different PCN throughout the course of the time period, as well as with the whole region to serve as a comparator of each PCNs with the average of the whole Wirral region. Spearman correlations were used for the association of HBPM utilization and i) rate of new hypertension diagnosis, ii) rate of BP control. Linear regression analysis was used to examine the relationship between HBPM use and i) rate of new diagnosis, ii) BP control; scatter plots with the regression line and residual plots were created.

## Results

A total number of 56.376 hypertensive patients in the Wirral CCG area were included in the analysis; of them, 10,010 provided an updated HBPM within the last 12 months. Participants were registered in 43 GP practices across Wirral area, corresponding to 5 different PCNs. Data were collected from January 2022 to September 2023. A summary of the results regarding the number of the study population, utilization of HBPM, new diagnoses of hypertension and achievement of BP control for this 20-month period by PCN and for the total population are reported in Table 1.

**Table 1:** Overview of hypertensives, rate of HBPM use, new hypertension diagnoses and control of BP in total Wirral area and per PCN.

PCN	Number of registered hypertensives	Number of hypertensives with a current HBPM	Rate of HBPM per 1000 hypertensives (95% CI)	Percentage of newly diagnosed hypertensives per month (%)	Percentage of BP control (95%CI)
PCN 1	21955	3014	14.36 (12.79; 16.07)	0.63	62.02 (60.98; 63.07)
PCN 2	8408	1555	24.62 (21.34; 28.26)	0.43	65.03 (63.32; 66.78)
PCN 3	13324	3410	26.76 (24.02; 29.72)	0.47	67.76 (66.37; 69.18)
PCN 4	3297	791	26.43 (20.99; 32.85)	0.73	63.42 (60.73; 66.2)
PCN 5	9392	1240	14.93 (12.52; 17.67)	0.54	64.57 (62.95; 66.21)
Wirral CCG	56376	10010	19.64 (18.48; 20.85)	0.55	64.33 (63.67; 65.00)

*BP* blood pressure, *HBPM* home blood pressure monitoring, *PCN* primary care network.

### HBPM utilization

**Fig. 1:**
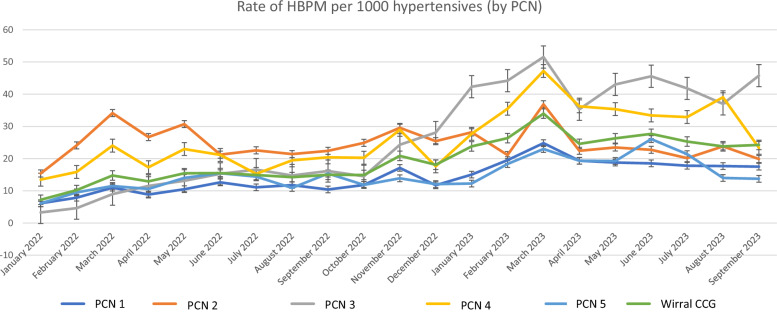
Rate of HBPM performed in each PCN per 1000 hypertensives from January 2022 until September 2023.

The average rate of HBPM per 1000 hypertensives differed significantly between the 5 PCN groups, as seen in Supplement Table 1. A graphical representation of HBPM utilization by PCN and for the total population in relation to time can be seen in Fig. 1. As depicted, there was a significant rise in use of HBPM for all PCN groups over time, with a greater increase after December 2022.

### New hypertension diagnoses

The average percentage of new hypertension diagnoses ranged from 0.43–0.73% per month between the different PCN (Table 1**)**. In line with the rise in HBPM utilization after December 2022, an increase in new hypertension diagnoses can be seen in the same time period (Supplement Fig. 1**)**; however, this effect was less transparent and not universal to all PCN groups.

### Blood pressure control

**Fig. 2:**
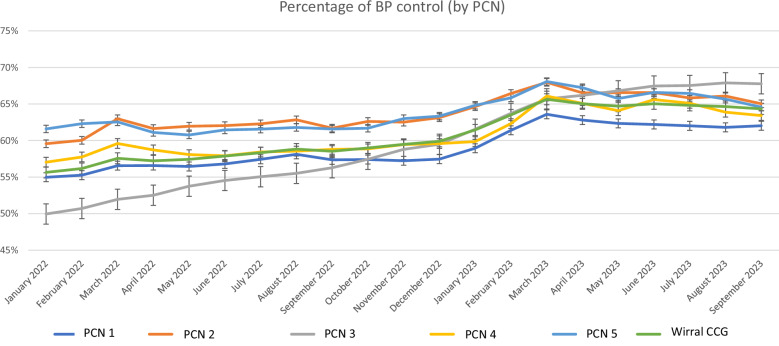
Percentage of BP control achievement (BP at target) in each PCN from January 2022 until September 2023.

Adequate BP control was achieved in the 64.3% of the total population on average during the 20 months of the study period (Table 1). Some PCNs accomplished a statistically higher rate of BP control, compared to others, however, these differences do not seem to reflect a clinically relevant magnitude (Fig. 2 and Supplement Table 2). During the study period, an increase of BP control was observed, being more noticeable after December 2022 for most PCN groups.

### Blood pressure diagnoses and control in relation to HBPM use

**Fig. 3:**
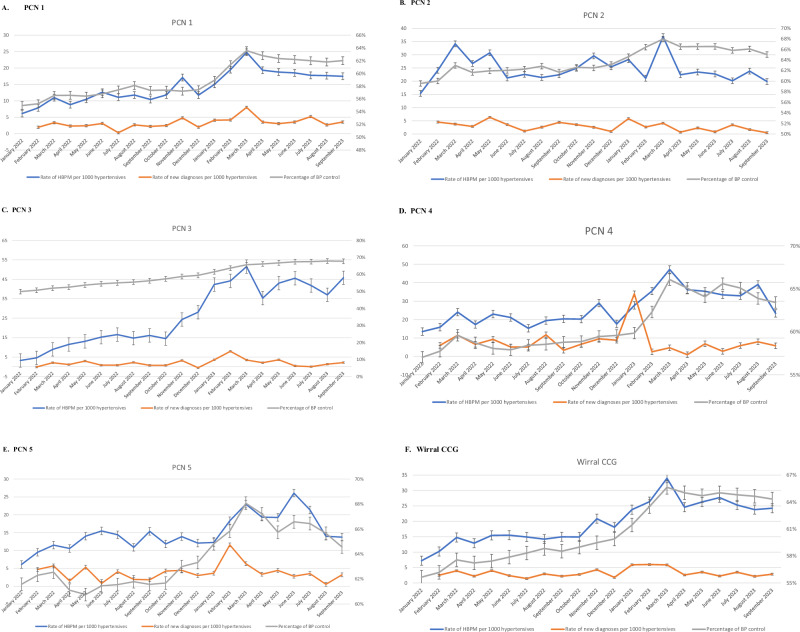
Rate of HBPM utilization, new hypertension diagnoses and percentage of BP control per month in each PCN and in total Wirral area; the scale on the right corresponds to the percentage of BP control. **A** PCN 1, **B**) PCN 2, **C**) PCN 3, **D**) PCN 4, **E**) PCN 5, **F**) Wirral CCG.

In order to examine the association of HBPM implementation with the rate of new diagnoses and rate of BP control, we plotted the aforementioned results in the same chart for the total population and for each PCN during the 20 months of the study duration (Fig. 3). It was observed that the use of HBPM and percentage of BP control display a parallel course through the months of the study period, while this relation is not readily distinct for the rate of new diagnoses by the plain inspection of the diagrams. To this end, we assessed spearman correlation coefficient for the association of HBPM use with new diagnoses and BP control rate. No significant correlation was observed for HBPM use and rate of new diagnoses (except for one PCN) (Table 2). However, utilization of HBPM was strongly associated with the achievement of BP control, as indicated by the high correlation coefficient for the majority of PCN and the total Wirral area (r = 0.914, p < 0.001 for Wirral area). Additionally, consistent results occurred in linear regression analysis (Table 3); significant association of HBPM use with BP control [B coefficient (95% CI): 0.48 (0.40–0.56), p < 0.001 for the total Wirral area] and a less consistent pattern of association with new hypertension diagnoses between different PCNs, but with a significant total association in the Wirral area [B coefficient (95% CI): 0.11 (0.02–0.20, p = 0.021]. The corresponding regression lines, depicting i) the association of rate of new hypertension diagnoses in relation to HBPM use can be found in Supplement Fig. 2; ii) the association of BP control in relation to the rate of HBPM utilization can be seen in Supplement Fig. 3 for each PCN and total Wirral area.

**Table 2:** Associations (spearman’s correlation coefficient) of rate of HBPM utilization with rate of new hypertension diagnosis and BP control in each PCN and in the total population.

PCN	Rate of new HTN diagnosis per 1000 registered	BP control
PCN 1	0.767**	0.892**
PCN 2	0.423	0.036
PCN 3	0.364	0.892**
PCN 4	−0.233	0.852**
PCN 5	0.069	0.578*
Wirral CCG	0.341	0.914**

*BP* blood pressure, *HBPM* home blood pressure monitoring, *PCN* primary care network, *HTN* hypertension.

*p < 0.01, **p < 0.001.

**Table 3:** Linear regression analysis of rate of HBPM utilization with rate of new hypertension diagnosis and BP control in each PCN and in the total population (data presented as B coefficient and 95% CI).

	Rate of new hypertension diagnosis per 1000 registered patients		BP control	
PCN	Beta coefficient (95% CI)	p value	Beta coefficient (95% CI)	p value
PCN 1	0.27 (0.16; 0.38)	**p** < **0.001**	0.54 (0.44; 0.65)	**p** < **0.001**
PCN 2	0.17 (0.01; 0.32)	**p: 0.036**	0.08 (−0.14; 0.31)	p: 0.461
PCN 3	0.05 (−0.01; 0.11)	p: 0.080	0.38 (0.32; 0.44)	**p** < **0.001**
PCN 4	−0.09 (−0.46; 0.29)	p: 0.634	0.29 (0.21; 0.37)	**p** < **0.001**
PCN 5	0.11 (−0.15; 0.37)	p: 0.400	0.37 (0.22; 0.52)	**p** < **0.001**
Wirral CCG	0.11 (0.02; 0.20)	**p: 0.021**	0.48 (0.40; 0.56)	**p** < **0.001**

Statistically significant p-values are bold.

*BP* blood pressure, *HBPM* home blood pressure monitoring, *PCN* primary care network, *CI* confidence intervals.

## Discussion

This study of over 10,000 hypertensive patients from the Wirral area, UK has shown that the implementation of a BP tele-messaging program with use of HBPM (initiated through BP@Home programme) increased the rate of new hypertension diagnoses and the rate of BP control. A considerable variation between different PCN groups was observed, in terms of new hypertension diagnoses and BP control achievement.

Different studies have identified the importance of HBPM in improving hypertension awareness and control. Moreover, HBPM can lead to identification of distinct phenotypes, such as white coat hypertension and masked hypertension, which would otherwise be undiagnosed by the mere use of office BP measurements [15]. In the era of digital health technologies, the implementation of BP tele-messaging systems has already arrived in clinical settings [23], representing a relatively simple and easy to adopt option [25]. Tele-transmission of BP measurements performed at home by patients has shown improvement in adherence to treatment, especially in combination with proper counselling [26], helping achieve better hypertension control rates [27–29] and improving cardiovascular prognosis [30, 31].

In our study, we identified an overall positive association between utilization of HBPM with tele-messaging and new diagnoses of hypertension, even though discrepancies occurred between different PCN groups. Most importantly, use of BP tele-messaging was strongly related to higher control rates of hypertension in this population. Differences between PCN groups regarding control rates were also observed. This effect might be attributed to better awareness of hypertension status and self-involvement by the patients, leading to higher adherence to treatment plans led by the physicians (either lifestyle interventions or antihypertensive medications). As noted above, certain discrepancies between different PCN groups could be explained by the different implementation rates of BP telemonitoring and differences in socioeconomic factors in each PCN; however, interpretation of this discrepancy was not the aim of the present analysis. Variation between PCNs may reflect differences in implementation intensity, practice engagement, and local population characteristics. Some practices more have more actively promoted BP@Home enrollment or had better digital workflows. These factors, though not quantifiable in this study, may have influenced the magnitude of BP control improvement.

The increasing adoption of HBPM in developed countries underscores the relevance of our findings beyond the population we studied. We acknowledge that variations in healthcare systems, patient education and cultural attitudes toward HBPM could influence the generalizability of our results. Therefore, we propose that future research explore these differences in diverse settings to better understand the broader applicability of our findings.

### Limitations

This is a pragmatic, real-life study of implementing a BP tele-messaging and telemonitoring system in a large area population. It was based solely on data collection using an automated tele-messaging program, therefore possible systematic biases regarding data selection from patients or data acquisition errors may have occurred. Moreover, no specific individual-level demographic information of the population or frequency of assessment for each patient could be collected due to the method of data acquisition, limiting the ability to correct our results for possible confounders, especially regarding digital engagement by older adults. Different confounders may have altered the rise in diagnosis and/or control of BP; however, no other specific campaigns or initiatives were applied in this region at the studied time point. Lastly, due to the observational nature of the study, no causative relationship could be established.

As a conclusion, implementation of a digital BP tele-messaging and telemonitoring HBPM programme in a large urban area is feasible and may lead to significant better control rates of hypertension, together with increase in diagnoses of new hypertension cases. Future studies should elucidate the cost-effectiveness and role of BP tele-messaging in lowering cardiovascular risk and achieving better outcomes in the general population.

## Summary

### What is known about topic:

- Home blood pressure monitoring (HBPM) may lead to better awareness and control of hypertension.
- The BP@Home programme supports the use of HBPM through a BP tele-messaging system with, offering a potential improvement in hypertension management.

### What this study adds:

- Utilization of a BP tele-messaging implemented approach with HBPM leads to increase in new hypertension diagnoses and better control of hypertension.
- Future studies should examine the role of BP tele-messaging with HBPM in lowering cardiovascular risk and improved outcomes in the community.

## Supplementary information

Supplement

## Data Availability

The datasets generated during and/or analysed during the current study are available from the corresponding author on reasonable request.
