# Supplementary material for: Effect of digital messaging on blood pressure control in general practice: observations from the BP@Home programme in wirral area
Source: J Hum Hypertens. 2025 Sep 25;39(11):770–6. doi: 10.1038/s41371-025-01072-y (PMC12592206; doi:10.1038/s41371-025-01072-y)

| **PCN** | **Average rate of HBPM per 1000 hypertensives (95% CI)** | **RR**  **(95% CI)** | **P value** |
| --- | --- | --- | --- |
| Wirral CCG  PCN 5  Wirral CCG | 19.64 (18.48; 20.85) | Reference | N/A |
| PCN 1 | 14.36 (12.79; 16.07) | 0.73 (0.64; 0.83) | **<0.001** |
| PCN 2 | 24.62 (21.34; 28.26) | 1.25 (1.08; 1.45) | **0.003** |
| PCN 3 | 26.76 (24.02; 29.72) | 1.36 (1.21; 1.54) | **<0.001** |
| PCN 4 | 26.43 (20.99; 32.85) | 1.35 (1.07; 1.68) | **0.009** |
| PCN 5 | 14.93 (12.52; 17.67) | 0.76 (0.63; 0.91) | **0.002** |
| HBPM, home blood pressure measurement; RR, rate ratio. | | | |

**Supplement Table 1.** Average rate and rate ratio of home blood pressure measurement among different sites in Wirral area**.**

**Supplement Table 2**. Rate of achieving blood pressure control per 100 hypertensives and rate ratio among different sites in Wirral area.

| **PCN** | **Rate of achieving BP control per 100 hypertensives (95% CI)** | **RR**  **(95% CI)** | **P value** |
| --- | --- | --- | --- |
| Wirral CCG | 64.33 (63.67; 65.00) | Reference | N/A |
| PCN 1 | 62.02 (60.98; 63.07) | 0.96 (0.95; 0.98) | **<0.001** |
| PCN 2 | 65.03 (63.32; 66.78) | 1.01 (0.98; 1.04) | 0.456 |
| PCN 3 | 67.76 (66.37; 69.18) | 1.05 (1.03; 1.08) | **<0.001** |
| PCN 4 | 63.42 (60.73; 66.20) | 0.98 (0.94; 1.03) | 0.525 |
| PCN 5 | 64.57 (62.95; 66.21) | 1.00 (0.98; 1.03) | 0.796 |

BP: blood pressure; RR: rate ratio.

**Supplement Figure 1**. Rate of newly diagnosed hypertensives per 1000 hypertensives per month in each PCN from February 2022 until September 2023.


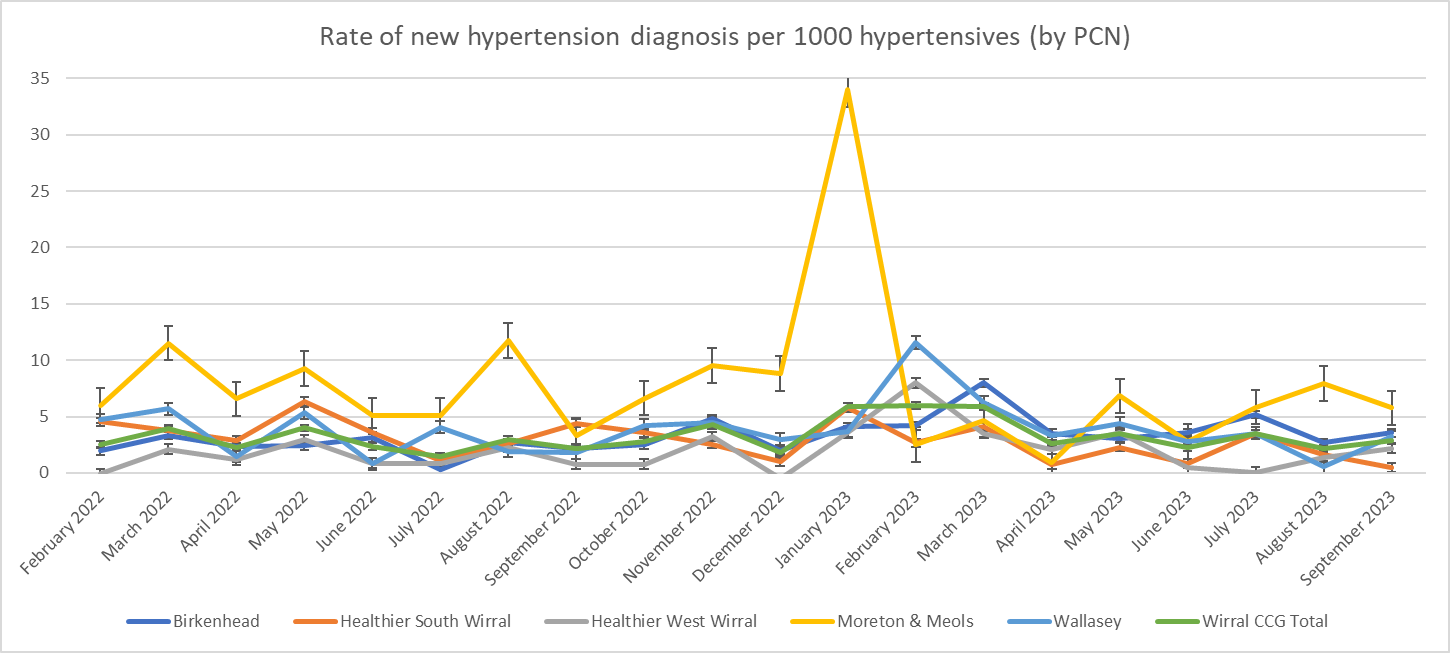


PCN 4

PCN 3

PCN 2

PCN 1

PCN 5

Wirral CCG

**Supplement Figure 2.** Regression lines of rate of HBPM utilization and rate of new diagnoses per 1000 hypertensives, per month for each PCN and for the total population.

1. **PCN 1**


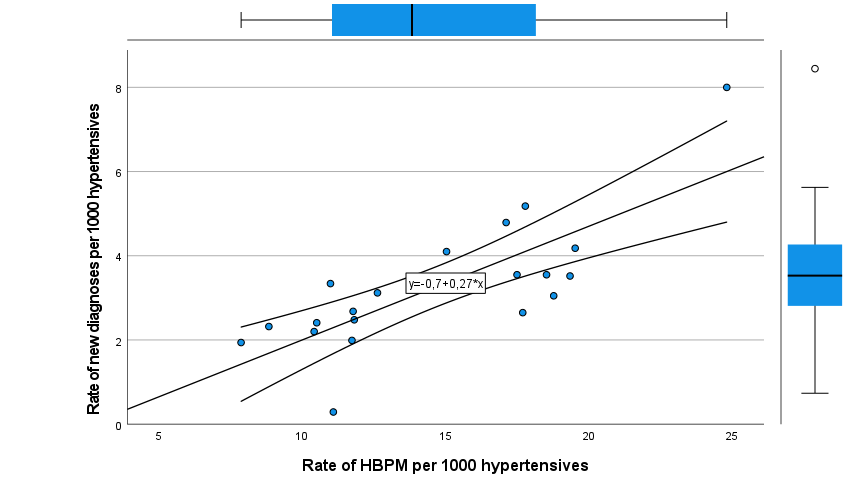


1. **PCN 2**


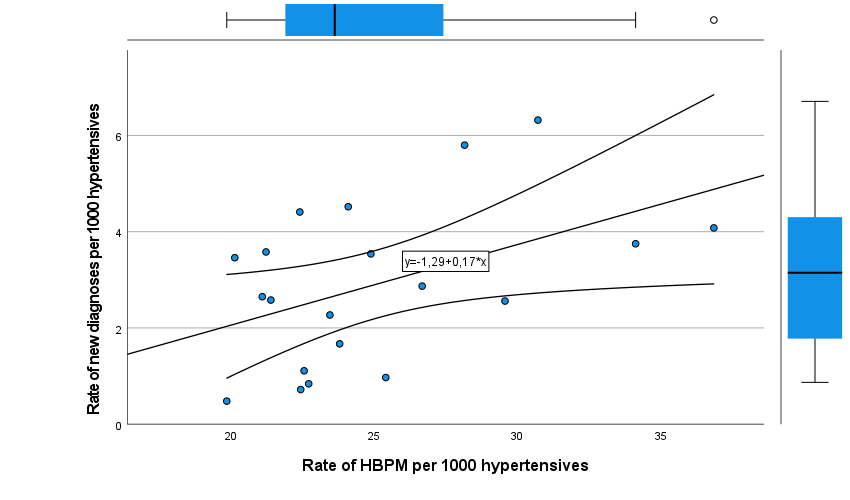


1. **PCN 3**


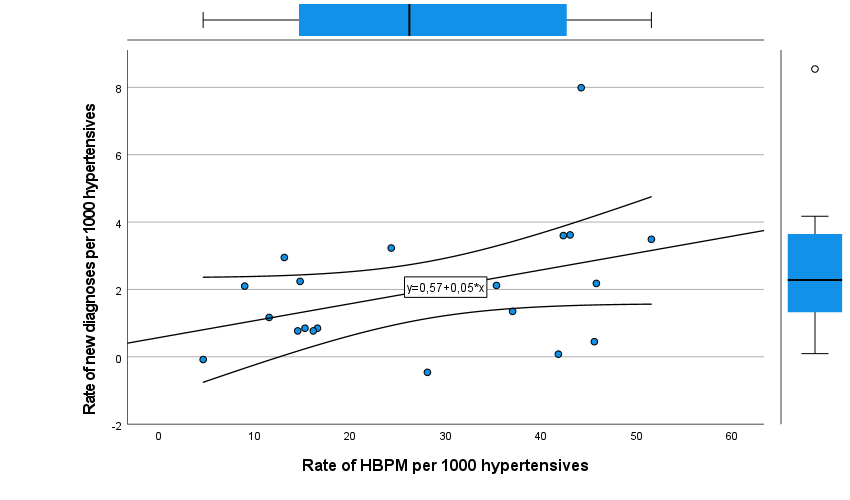


1. **PCN 4**


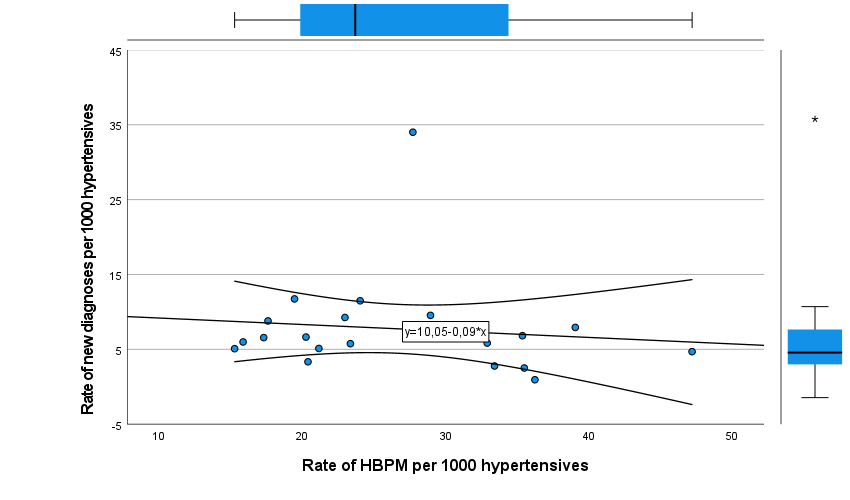


1. **PCN 5**


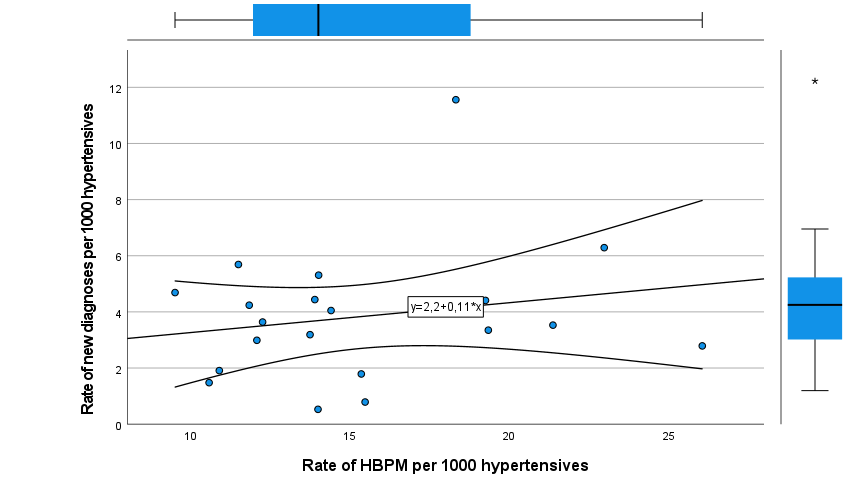


1. **Wirral CCG**


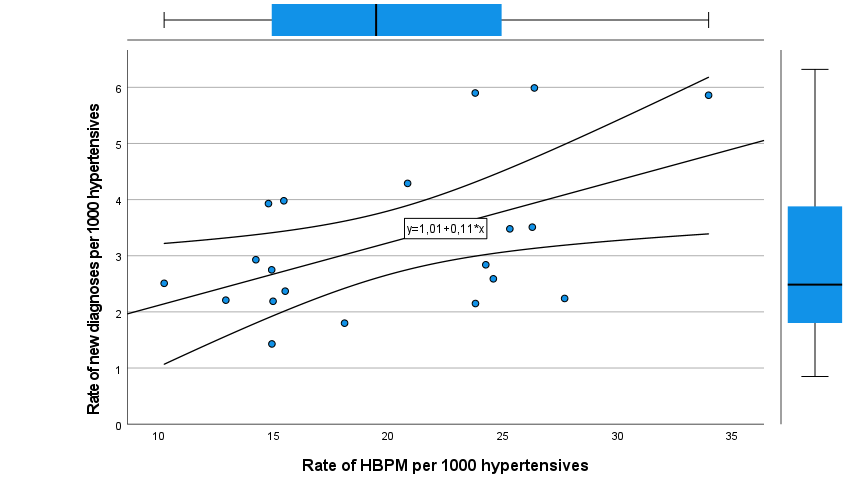


**Supplement Figure 3.** Regression lines of rate of HBPM utilization and BP control, per month for each PCN and for the total population.

1. **PCN 1**


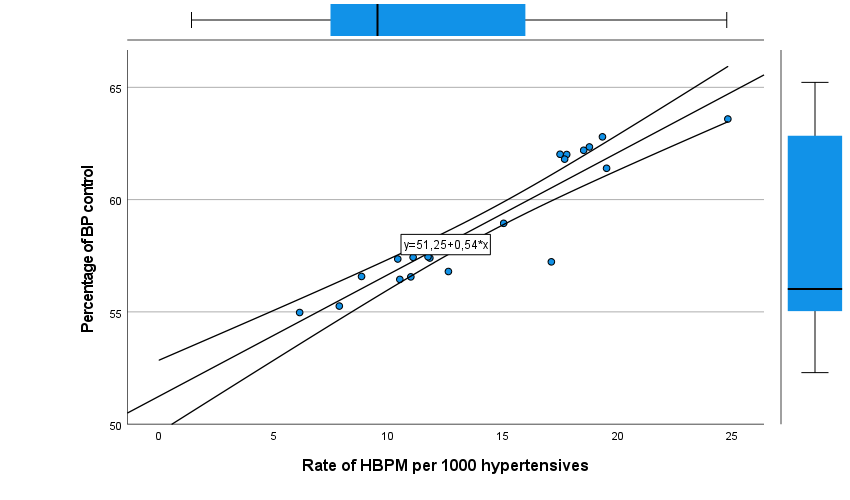


1. **PCN 2**


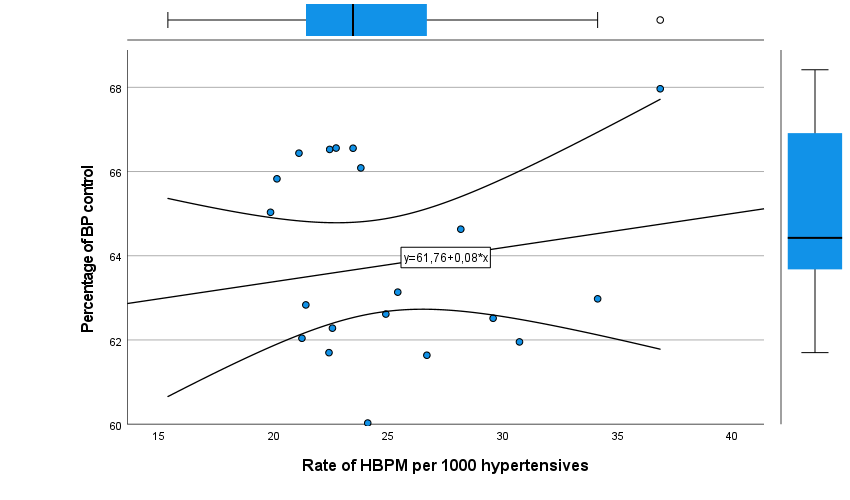


1. **PCN 3**


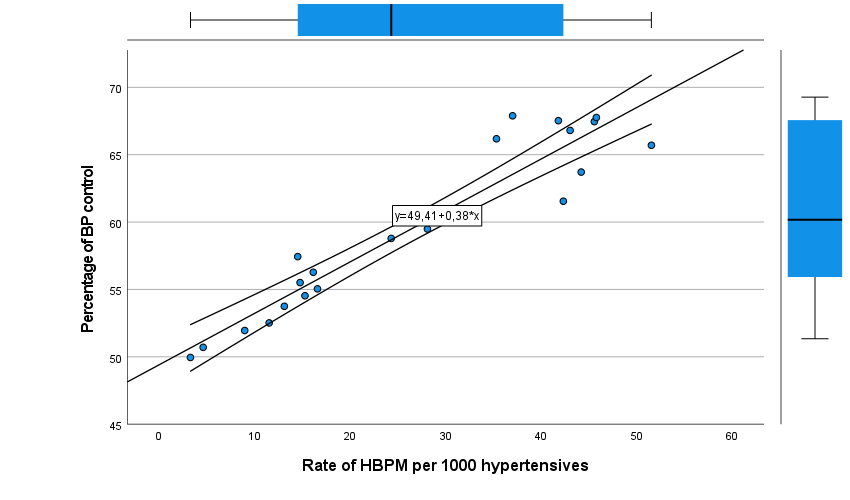


1. **PCN 4**


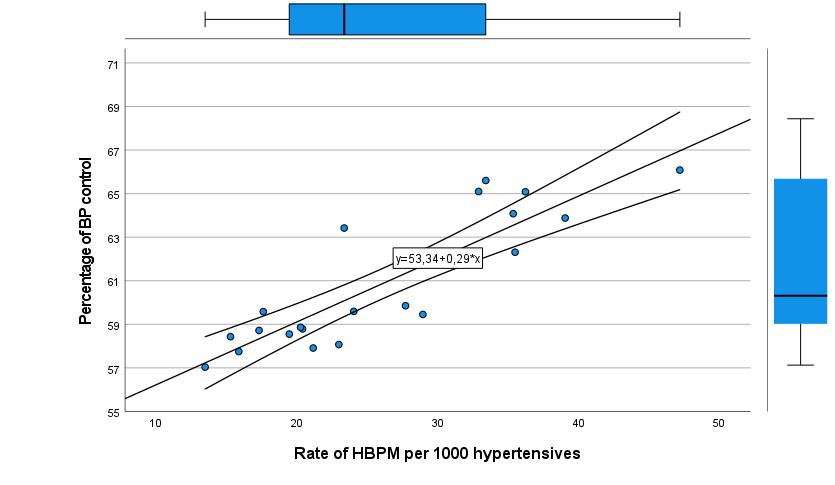


1. **PCN 5**


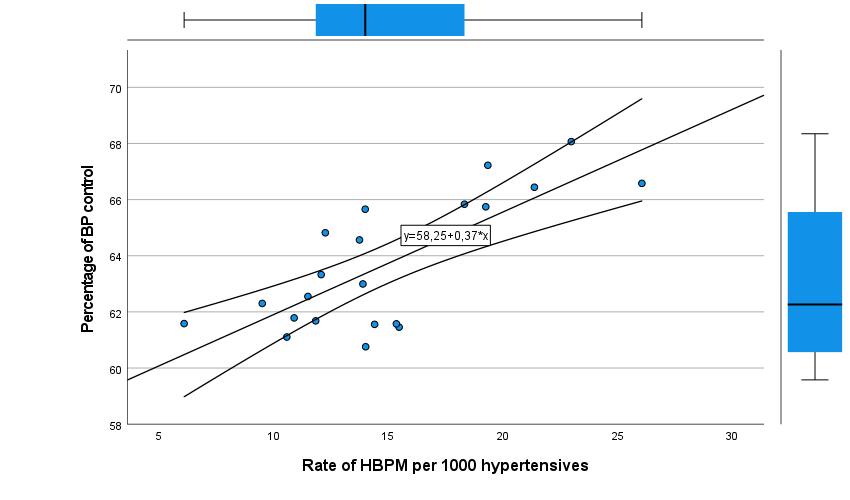


1. **Wirral CCG**


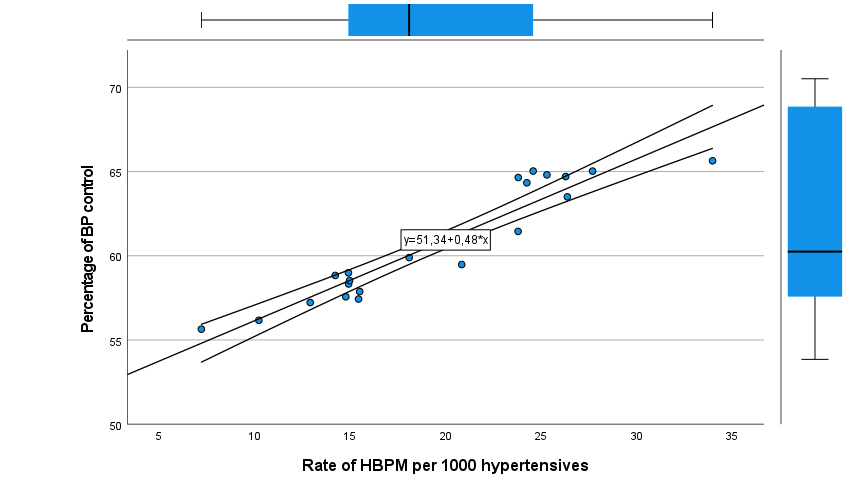

Supplement: Supplementary file 1 — Supplement [file 41371_2025_1072_MOESM1_ESM.doc]
